# Suppressing evolution in genetically engineered systems through repeated supplementation

**DOI:** 10.1111/eva.13119

**Published:** 2020-11-06

**Authors:** Nathan C. Layman, Beth M. Tuschhoff, Andrew J. Basinski, Christopher H. Remien, James J. Bull, Scott L. Nuismer

**Affiliations:** ^1^ Department of Biological Sciences University of Idaho Moscow ID USA; ^2^ Department of Mathematics University of Idaho Moscow ID USA

**Keywords:** bioreactor, gene drive, gene flow, genetic engineering, swamping, transmissible vaccine

## Abstract

Genetically engineered organisms are prone to evolve in response to the engineering. This evolution is often undesirable and can negatively affect the purpose of the engineering. Methods that maintain the stability of engineered genomes are therefore critical to the successful design and use of genetically engineered organisms. One potential method to limit unwanted evolution is by taking advantage of the ability of gene flow to counter local adaption, a process of supplementation. Here, we investigate the feasibility of supplementation as a mechanism to offset the evolutionary degradation of a transgene in three model systems: a bioreactor, a gene drive, and a transmissible vaccine. In each model, continual introduction from a stock is used to balance mutation and selection against the transgene. Each system has its unique features. The bioreactor system is especially tractable and has a simple answer: The level of supplementation required to maintain the transgene at a frequency $\hat{p}$ is approximately $\hat{p}s$, where *s* is the selective disadvantage of the transgene. Supplementation is also feasible in the transmissible vaccine case but is probably not practical to prevent the evolution of resistance against a gene drive. We note, however, that the continual replacement of even a small fraction of a large population can be challenging, limiting the usefulness of supplementation as a means of controlling unwanted evolution.

## 1 INTRODUCTION

The tools provided by genetic engineering can fundamentally alter our technological approaches to medicine, agriculture, and ecology. Through their use, crops have been designed to increase yield while reducing loss from pests and disease (Pellegrino, Bedini, Nuti, & Ercoli, 2018). Critical medications, such as insulin and the mammalian growth hormone‐inhibiting hormone, are now produced using bio‐engineered organisms (Itakura et al., 1977). Genetically engineered organisms have been proposed to help facilitate adaptive responses to climate change, suppress undesirable or invasive populations, and reverse the fixation of deleterious mutations in at‐risk populations of endangered plants and animals (Thomas et al., 2013). In addition, genetic engineering has made vaccines safer, more effective, faster to produce, and could make them easier to disseminate (Basinski et al., 2018; Chahal et al., 2016).

Although promising, these new technologies all confront a common challenge: unwanted evolution that may undermine the intent of engineering. Transgenes, stretches of foreign DNA inserted into a host genome, evolve in the same way as any other genetic material. Consequently, if a transgene compromises fitness, selection favors its potentially rapid removal from the population (Sleight, Bartley, Lieviant, & Sauro, 2010; Willemsen & Zwart, 2019). Even when the insert does not reduce fitness, mutations can accumulate, leading to loss of function. This “evolutionary half‐life” poses a significant challenge for the design of successful genetically modified organisms and motivates the identification of designs that slow or arrest evolution of genetic modifications (Bull & Barrick, 2017).

One possible solution to suppress evolution in genetically modified organisms comes from population genetics theory demonstrating that gene flow can impede local adaptation (Akerman & Bürger, 2014; Bolnick & Nosil, 2007; Crow & Kimura, 1970; Haldane, 1957; Lenormand, 2002; Levene, 1953; Slatkin, 1973; Wright, 1940). In the classic “mainland‐island” models, migration from a mainland to an island can suppress island adaptation. By suppressing local adaptation, immigration can maintain costly phenotypes that would otherwise be displaced by evolution. An interesting question is whether “migration” can be used to maintain essential functions of genetically modified populations in the face of countering selection. In this context, repeated re‐introduction of genetically modified individuals into an evolving population is analogous to a constant level of immigration from a source fixed for locally unfavorable mutations.

To address this question, we develop and analyze mathematical models to evaluate the efficacy of repeated introduction (henceforth “supplementation”) as a tool for controlling unwanted evolution in three contexts of genetically engineered populations: bioreactors, gene drives, and transmissible vaccines. In the first, we identify the level of supplementation required to maintain a stable frequency of a desired but deleterious transgene in the microbial population of a bioreactor. In the second, we evaluate the effect of supplementation on the spread and stability of gene drives that experience resistance evolution. In the third, we identify the amount of supplemental direct vaccination required of a transmissible vaccine to prevent its evolutionary decay while maintaining a high enough vaccine coverage to also protect the population against a pathogen.

## 2 THE MODELS

### 2.1 Maintaining engineered genes in a bioreactor

Approaches used to grow and maintain transduced cell populations in bioreactors can be broadly classified as batch culture and continuous. In batch culture methods, a bioreactor is filled with the appropriate media, inoculated and left to run for a fixed amount of time or until production of the transgene product falls below a threshold. During the initial stage, resources are not limited, and the population of cells grows rapidly. Eventually, however, growth rates slow as resources become depleted, resulting in cell quiescence or population collapse. Once this occurs, the culture is harvested, and the process starts again. In continuous culture methods, for example, chemostats, some fraction of the bioreactor is continually replaced with fresh media. In this way, growth can be maintained and even controlled by varying the dilution rate. In addition to their use in the production of industrial products such as insulin, bioreactors of both kinds have been used extensively in the study of evolution.

Bioreactors have many advantages for evolutionary studies including large population sizes and fast generation times. However, these advantages can complicate production because of unwanted evolution. For example, Gresham and Hong (2015) estimated that in a standard chemostat, the mutational supply rate is high enough to, each generation, introduce every possible point mutation along the entire genome of *S. cerevisiae*, an organism commonly used in the production of biopharmaceuticals such as insulin. Some of these mutations would disable the transgene (Dietz‐Pfeilstetter, 2010; Kazemi et al., 2013; Rajeevkumar, Anunanthini, & Sathishkumar, 2015; Sleight et al., 2010; Springman, Molineux, Duong, Bull, & Bull, 2012). Furthermore, evidence suggests that costs imposed by transgenesis are both common and difficult to predict (Schmerer et al., 2014; Sleight et al., 2010). Engineering transgenes that minimize fitness costs or mutation rates can delay the spread of unwanted mutations (Sleight et al., 2010), but the ability to engineer such transgenes may be limited by fundamental metabolic or physical constraints. When this is true, arresting undesirable evolution is possible through mechanisms such as supplementation (Bull & Barrick, 2017).

We start by modeling a culture of haploid individuals in a continuously cultured bioreactor. The engineered transgene is designated *A*. We use this model to investigate how a transgene can be maintained at high frequency in a bioreactor, despite a selective disadvantage. Mutation erodes *A* at a rate of *μ* mutations per generation, converting it to a degraded form (designated *a*). Without supplementation, the forces of selection and mutation will ensure the eventual loss of *A*. We assume the reactor is well mixed and that population sizes are sufficiently large for the impacts of genetic drift to be ignored. Time consists of discrete nonoverlapping generations. During each generation, the life cycle consists of (a) selection, (b) mutation, and (c) supplementation. Selection is assumed to result from a reduced growth rate of the engineered genotype imposed by transcription or translation of the transgene, although other mechanisms of inference are possible. Individuals expressing the functional transgene (*A*) therefore experience a relative cost in growth (*s*) compared to those expressing degraded (*a*) alleles. Mutation is assumed to be unidirectional from *A* to *a*, as would be expected for disruptions of a transgene, where back mutation to a functional transgene is extremely unlikely. Supplementation involves the replacement every generation of a fraction of the population, *σ*, with a bolus that is comprised primarily of the engineered genotype *A*, as well as a small fraction of mutant individuals *a* (which will be unavoidable in most implementations). Together, these assumptions lead to the following recursion for the frequency of the transgene over one generation:

(1) $$\begin{array}{c} p^{\prime }=\frac{p\left(1‐s\right)\left(1‐\mu \right)}{1‐ps}\left(1‐\sigma \right)+\sigma \left(1‐\mu \right), \end{array}$$

where parameters and variables are defined in Table 1. This recursion can be used to identify the critical level of supplementation, $\sigma_{C}^{*}$, required to maintain *A* at some desired frequency $\hat{p}$:

(2) $$\begin{array}{c} \sigma_{C}^{*}=\frac{\hat{p}s\left(1‐\hat{p}\right)+\hat{p}\mu \left(1‐s\right)}{\left(1‐\hat{p}\right)\left(1‐\mu \right)}\approx \hat{p}s. \end{array}$$

**TABLE 1:** Bioreactor model of haploids

Genotype	*A*	*a*
Frequency	*p*	1 − *p*
Fitness	1 − *s*	1
Mutation rate	*μ* (*A* → *a*)	0 (*a* → *A*)
Supplementation level	*σ* (1 − *μ*)	*σ* *μ*

**FIGURE 1:**
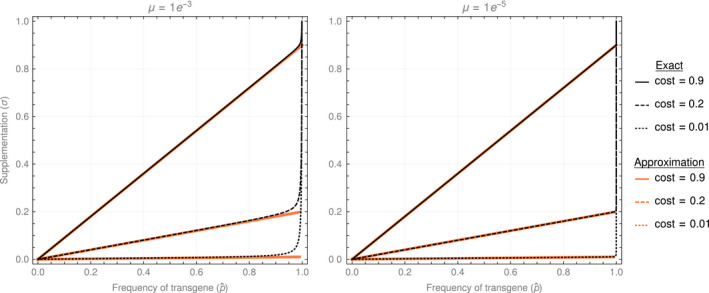
The level of per‐generation supplementation (*σ*) required to maintain an equilibrium frequency (p*) of a transgene with a selective cost of *s* in a chemostat bioreactor. The orange curves represent the approximation *σ* ≈ *p* s. Mutation rates (*μ*) are per generation and were chosen to highlight the differences between the exact and approximate solutions at extremes, but the rates also span potentially reasonable values under different engineering designs (Sleight et al., 2010; Williams, 2014)

To a first approximation, Equation (2) reveals that the level of supplementation required to maintain stability increases as a function of selection acting against the transgene and the desired frequency of the transgene. Consequently, the only way to maintain a transgene in a bioreactor at high frequency without large‐scale supplementation is with a low cost of the transgene (Figure 1). As this is an equilibrium result, the mutation rate has little effect—provided it is low and there is no attempt to maintain the transgene near a frequency of 1; mutation rate affects the maintenance of the transgene at very high frequencies in part because the inoculum itself is increasingly contaminated by mutants (compare panels in Figure 1). On the other hand, while it serves as the source of the selected variation, mutation itself does not require much additional effort to offset (Figure 1; red vs. black curves).

These results demonstrate that even moderately weak selection by engineering standards can be a major obstacle for maintaining high frequencies of a transgene. For example, maintaining the transgene in half the population when *s* = 0.05 requires replacement of 2.5% of the population per generation. With bacterial generation times of half an hour, this translates into replacing more than the bioreactor volume per day, very possibly not practical. Furthermore, any stock serving as the source of supplementation will face the same problem experienced by the bioreactor—selection—and thus may have a much higher frequency of type *a* than given by the mutation rate. This latter problem can potentially be mitigated by growing stock cultures used for replacement in an environment that silences expression of the transgene and so avoids selection.

Another approach to minimize the effects of mutation and selection is to start a bioreactor at a high transgene frequency and allow it to decay, dumping the media and starting over once production drops below some threshold—as in the batch culture method. This could be an attractive approach when the system only needs to maintain a high frequency for a short period of time or when continual supplementation is impractical. Here, in addition to the eventual outcome, the speed of decay is an important design consideration. Solving Equation (1) (without supplementation, *σ* = 0) for the frequency of *A* at time *t* yields:

(3) $$\begin{array}{c} p_{t}=\frac{sp_{0}+\left(1‐s\right)\mu p_{0}}{sp_{0}+{\left(\frac{1}{\left(1‐s\right)\left(1‐\mu \right)}\right)}^{t}\left(\mu +s\left(1‐\mu ‐p_{o}\right)\right)}. \end{array}$$

**FIGURE 2:**
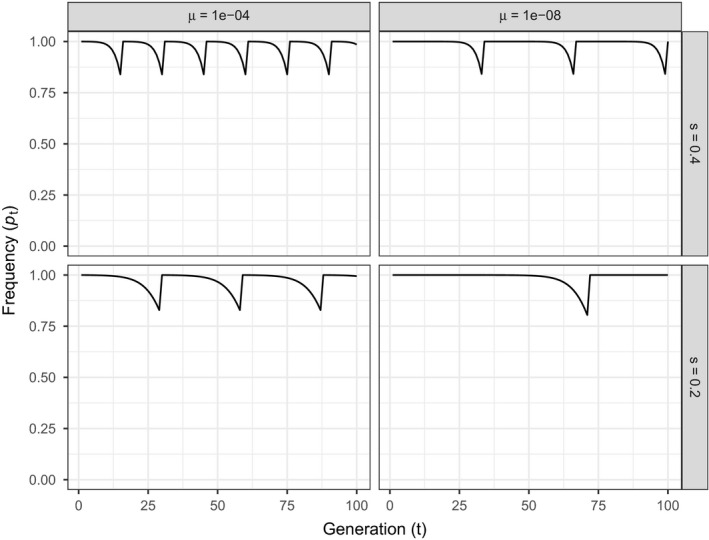
The strategy of bioreactor supplementation by replacement of the entire culture when transgene frequency (*p*) drops below a threshold. In each of the four panels, Equation 3 was initialized with a starting frequency (*p*_0_) of 1 − *μ* and time advanced until *p* fell below 0.8 In the following generation, the culture was discarded and replaced with a new population at the same starting frequency as the first trial. Panels within a row vary mutation rate; panels within a column vary selection (disadvantage of transgene carriage)

Figure 2 shows the frequency of the transgene over time in a batch culture bioreactor initialized with a starting frequency ($p_{0}$) of 1 − μ and subject to periodic purging and re‐initiation. During re‐initiation, $p_{t}$ is briefly reset to $p_{0}$ whenever the transgene falls below a threshold frequency of 80%. Here, the benefit of minimizing mutation rate as well as a fitness cost is clear—reducing the mutation rate slows the decline of a transgene even when it has little influence on final equilibrium conditions. Whether this form of episodic supplementation is feasible will depend on the relative merits of less frequent supplementation versus the impact of larger declines in the frequency of *A*.

Overall, supplementation of some form (which can encompass the extreme of total population replacement) becomes an essential component of any protocol in which the selective cost of engineering is as high as even a few percent unless mutation rates to loss of function are extremely small (e.g., less than 10^–8^). It should also be appreciated that our forecasts are deterministic; when population sizes are small, the ascent of nonfunctionality may be delayed substantially.

### 2.2 Delaying the evolution of resistance to gene drives

Gene drives are constructs designed to take advantage of non‐Mendelian segregation (or biased survival). One type of gene drive (a “modification” drive) can be used to rapidly spread transgenic DNA throughout a population (for a review, see Champer, Buchman, & Akbari, 2016). Another type of engineered gene drive (a “suppression” drive) is designed for population suppression (Kyrou et al., 2018). A central challenge of using gene drives for suppression is that resistance is favored to block the drive. Indeed, resistance mutations can fully undermine population suppression efforts in the long term (Bull, 2017; Burt, 2003; Deredec, Burt, & Godfray, 2008; Hammond et al., 2017; Unckless, Clark, & Messer, 2017). Here, we utilize a single‐locus, three‐allele model to determine whether gene drive supplementation might be effective in reducing the impact of resistance evolution. This differs from the bioreactor model in that supplementation is used here to prevent the evolution of resistance against a genetically modified organism rather than in the organism itself.

The model design is given in Tables 2 and 3. We assume a large, panmictic population in which mating is followed by selection followed by mutation. The wild‐type allele *W* is vulnerable to distortion by a drive allele *D*. Thus, in heterozygotes with the wild‐type allele (*DW*), *D* displaces a fraction *c* of *W* bearing gametes with itself—biasing segregation in its own favor. Genotype *DD* suffers a fitness cost (*s*), with possible costs extended to heterozygotes carrying *D* (*hs*), depending on the degree of dominance (*h*). Mutation of *W* generates resistant alleles *R* at a constant rate, *μ*. *DR* heterozygotes segregate normally (Mendelian).

**TABLE 2:** Gene drive model allele frequencies

	Allele	Frequency
Driving	*D*	*d*
Wild‐type	*W*	*w*
Resistant	*R*	*r*

**TABLE 3:** Gene drive model genotypes and parameters

Genotype	Frequency	Fitness	Seg. bias
*DD*	*d* ^2^	1 − *s*	
*DW*	*2dw*	1 − *hs*	*(c + 1) D: (1 − c) W*
*DR*	*2dr*	1 − *hs*	*1 D: 1 R*
*WW*	*w* ^2^	1	
*WR*	*2wr*	1	*1 W: 1 R*
*RR*	*r* ^2^	1	

While it is also possible that resistant mutants arise as a result of failed attempts to convert wild‐type alleles, as when the drive mechanism is a homing endonuclease, we have chosen instead to focus on de novo mutations that confer resistance. The major difference is that de novo mutations arise before the drive begins to spread. Resistance alleles formed by imperfect conversion would only accrue after the drive reaches a high frequency, when supplementation would be least effective (see Unckless et al., 2017 for more detail on the breakdown of gene drives). Our model is designed to capture the dynamics of population suppression gene drives (large *s*) but can also be applied to cargo drives that attempt to modify populations (low *s*) without causing extinction (Hammond et al., 2016; Lambrechts, Koella, & Boëte, 2008). In these circumstances, resistance arising from failed drive conversion may play a larger role.

To supplement the drive, a fixed proportion of *DW* individuals (*σ*) are introduced following mutation. If viable, *DD* homozygotes could be used to supplement the drive instead of heterozygous *DW* individuals, but we consider this assumption as impractical for most suppression drives which are designed to have very large *s*, possibly to the extreme of *DD* being sterile or inviable. Furthermore, if gene drive is extreme, a *DW* individual will produce nearly all *D* gametes (we consider supplementation by *DD* in the Appendix 1).

Together, these assumptions yield the following set of recursion equations for the frequency of the wild‐type (frequency *w*) and resistant (frequency *r*) alleles:

(4) $$\begin{array}{c} w^{\prime }=\frac{\left(w\left(1‐cd‐dhs\left(1‐c\right)\right)+\frac{\sigma }{2}\left(1‐ds\left(d+2h\left(1‐d\right)\right)\right)\left(1‐c\right)\right)\left(1‐\mu \right)}{1‐ds\left(d+2h\left(1‐d\right)\right)}, \end{array}$$

(5) $$\begin{array}{c} r^{\prime }=\frac{\left(r\left(1‐dhs\right)+\mu w\left(1‐cd‐dhs\left(1‐c\right)\right)\right)\left(1‐\sigma \right)+\frac{\sigma \mu }{2}\left(1‐ds\left(d+2h\left(1‐d\right)\right)\right)\left(1‐c\right)}{1‐ds\left(d+2h\left(1‐d\right)\right)}. \end{array}$$

**FIGURE 3:**
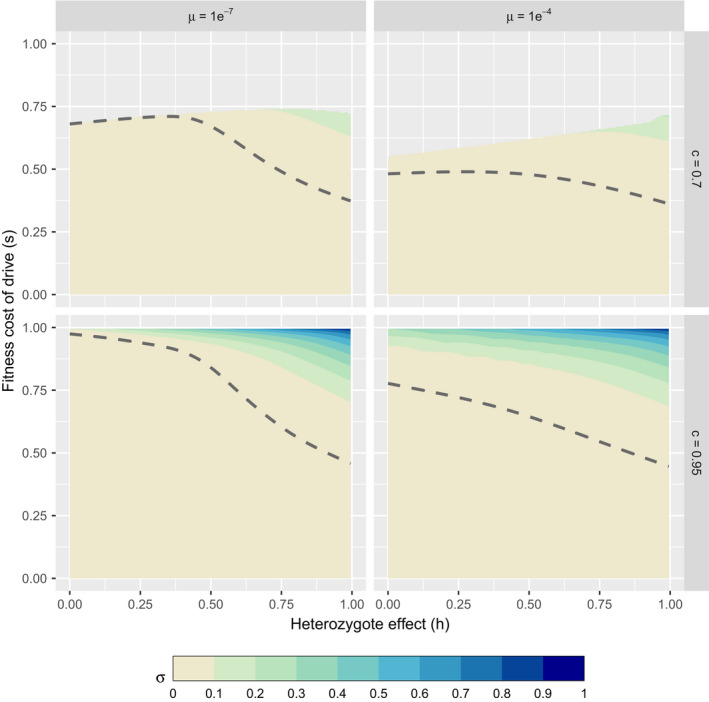
Supplementation necessary to achieve a peak gene drive allele frequency of ≥95% for differing mutation and drive conversion efficiencies (c). Text Equations (4) and (5) were iterated across 100 generations assuming a starting frequency of 1 × 10^−6^ for both the gene drive and resistance mutations. The hashed area delineates parameter combinations where the drive fails to reach the target frequency regardless of supplementation effort. The dashed lines show the boundary region above which nonzero supplementation was required for the drive to reach the target threshold

We investigated the magnitude of supplementation of the *DW* genotype required to attain a target frequency of 95% *D* (Figure 3); note that the 95% may be a temporary frequency, not an equilibrium. The results necessarily depend on all parameter values, and only a small set is considered here. Over most of the parameter space considered, *D* requires little supplementation to reach the target frequency. This is unsurprising given that gene drives are designed to spread rapidly and autonomously and that the evolution of resistance has been shown to be insensitive to drive starting frequency even when mutation degrades the drive itself instead of imbuing conversion resistance to wild‐type alleles (Unckless et al., 2017). It is only when the fitness of drive homozygotes is large (*s* ≈ 1) or when the heterozygote suffers nearly the same fitness cost as the *DD* homozygote (*h *≈ 1) that supplementation becomes relevant. Engineering should often be able to avoid high values of *h* entirely, if desired (Champer, Zhao, Champer, Liu, & Messer, 2020).

**FIGURE 4:**
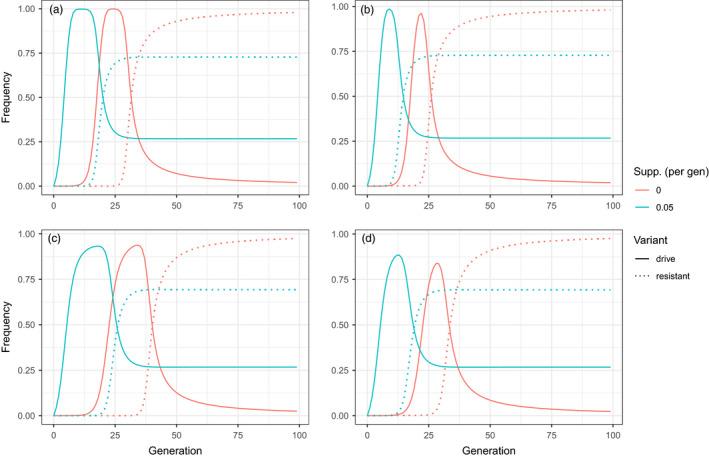
Supplementation to avert evolution of resistance to a gene drive has little effect on the maximum frequency attained by a gene drive, but it hastens gene drive evolution and suppresses final resistance allele frequency. The frequency of both drive and drive‐resistant alleles over time is shown, obtained by iterating Equations (4) and (5) for 100 generations. In the top row (panels a and b), the gene drive efficiency was set to 0.95; in the lower row (panels c and d), it was set to 0.7. The mutation rates from wild‐type to resistant alleles were 1 × 10^−7^ in the first column (a,c) and 1 × 10^−4^ in the second column (b, d). These numbers were chosen from previously estimated mutation rates for a gene drive in *Drosophila melanogaster* (10^–4^ to 10^–8^) and span decay rates (0.6 * 10^–6^ to 1.7 * 10^–6^) of inserted *lacI* in transgenic mice (Edgington & Alphey, 2019; Kohler et al., 1991). In all cases, the selection acting against drive homozygotes was s = 0.7. Simulations were started with the resistant allele already present in the population at a frequency of 10^–7^

To investigate whether supplementation can delay the evolution of resistance, we numerically iterated Equations (4) and (5) for a drive whose fitness effect on the individual is fully recessive (Figure 4). If supplementation can delay the spread of resistance, it might be possible to bring a population to a low enough density rapidly enough to result in stochastic extinction before resistance evolves (Burt, 2003). Inspection of the figure reveals that the two main effects of supplementation are to (a) hasten the ascent of the drive and (b) suppress the frequency of the resistance allele. While the latter effect is a straight‐forward consequence of ongoing supplementation, this technique does not appear to be a useful tool to increase the maximum frequency of a gene drive.

### 2.3 Supplementation to offset evolution of transmissible vaccines

A classical finding from epidemiology is that vaccine coverage must exceed a threshold for a pathogen to be eliminated from a population (Anderson & May, 1979; Keeling & Rohani, 2008). This threshold is determined by several parameters, including the effectiveness of the vaccine, the duration of immunity, and the transmissibility of the pathogen. Many common pathogens, such as measles and pertussis, require that a large fraction of the population be vaccinated to eradicate a population from disease (Fine, 1993). However, high coverage can be difficult to achieve with traditional vaccines and, once reached, challenging to maintain.

Transmissible vaccines reduce the level of direct vaccination necessary to achieve pathogen eradication (Nuismer, Basinski, & Bull, 2019). These novel vaccine designs offer new opportunities for eliminating the threat of many zoonotic pathogens by targeting them in their wild animal reservoirs before they disseminate into human populations. One blueprint for designing a transmissible vaccine is to insert an immunogenic transgene derived from a specific pathogen into a fully competent but benign “vector” virus. This approach is attractive because infection with a transgenic vector can then promote an immune response against a pathogen without any risk of disease or of evolution to regenerate the wild‐type pathogen. However, the transgene is expected to experience evolutionary decay unless it provides a benefit to the vector (Bull, Nuismer, & Antia, 2019). Even if the “intrinsic” cost of carrying an antigenic transgene can be completely avoided, mutations that reduce immune overlap between the vaccine and the pathogen will still be favored, reducing the effectiveness of a transmissible vaccine (Basinski et al., 2018; Bull, Smithson, & Nuismer, 2018; Evans et al., 1985; Nuismer et al., 2019).

Fundamentally, vaccination is a process of supplementation. Calculations of herd immunity determine the level of supplementation of a nonreplicating vaccine necessary to keep a pathogen from invading. Previous work on transmissible vaccines has shown that the level of direct vaccination necessary to protect a population can be greatly reduced using even weakly transmissible vaccines, but they are subject to evolutionary decay (Basinski et al., 2018; Nuismer et al., 2019). Here, we expand on these models to ask whether the effectiveness of a transmissible vaccine subject to evolutionary decay can be maintained in the face of selection by continual supplementation.

As with previous models of transmissible vaccine evolution, we assume that the mutated/degraded vaccine strain is initially absent from the population. The mutated version of the vaccine is considered phenotypically equivalent to the untransformed, or “empty” vector. We therefore assume complete cross‐immunity so that individuals exposed to the vaccine are immune to infection by the vector and individuals exposed to the vector are immune to the vaccine. Previous studies have suggested that rare vector serotypes be chosen as vaccine platforms to avoid the problem of pre‐existing vaccine immunity due to exposure to the vector (Lasaro & Ertl, 2009; Rollier, Reyes‐Sandoval, Cottingham, Ewer, & Hill, 2011; Saxena, Van, Baird, Coloe, & Smooker, 2013). To that end, we also assume that the specific vector strain capable of competing with the vaccine is initially absent in the population and only introduced through mutation (see Basinski et al., 2018 for more detail on cross‐immunity). Together, these assumptions yield the following system of differential equations describing the change in the densities of susceptible hosts (*S*), vaccine‐infected hosts (*V*), degraded vaccine‐infected hosts (*W*), hosts recovered from vaccine infection (*R_V_*), and hosts recovered from infection by the degraded vaccine (*R_W_*):

(6) $$\begin{array}{c} \dot{S}=b\left(1‐\sigma \right)‐\beta_{V}SV‐\beta_{W}SW‐dS, \end{array}$$

(7) $$\begin{array}{c} \dot{V}=b\sigma +\beta_{V}SV‐\gamma_{V}V‐\mu V‐dV, \end{array}$$

(8) $$\begin{array}{c} \dot{W}=\beta_{W}SW‐\gamma_{W}W+\mu V‐dW, \end{array}$$

(9) $$\begin{array}{c} {\dot{R}}_{V}=\gamma_{V}V‐dR_{V}, \end{array}$$

(10) $$\begin{array}{c} {\dot{R}}_{W}=\gamma_{W}W‐dR_{W}. \end{array}$$

**FIGURE 5:**
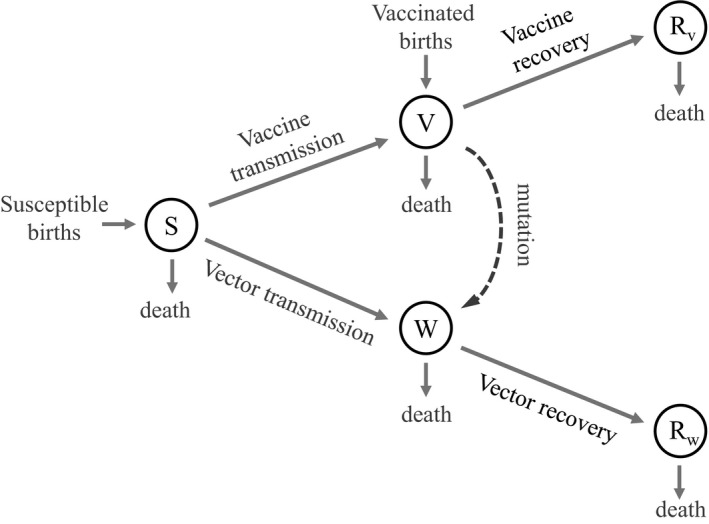
Susceptible–infectious–recovered (SIR) model flow chart for a transgenic transmissible vaccine with antigenic decay. This figure accompanies text Equations (6)–(10)

The model structure is outlined in Figure 5, and all parameters and variables are defined in Table 4. Our results are presented in terms of vaccine (and pathogen) basic reproductive numbers (*R*_0_). Pathogen *R*_0_ is merely a given, but the vaccine *R*_0_ is calculated as: $\left(\frac{b}{d}\right)\left(\frac{\beta_{V}}{d+\mu +\gamma_{V}}\right)$.

**TABLE 4:** Transmissible vaccine model parameters

Variable	Description
S	Susceptible hosts (density)
V	Vaccine‐infected hosts (density)
W	Degraded vaccine‐infected hosts (density)
$R_{V}$	Hosts recovered from vaccine infection (density)
$R_{W}$	Hosts recovered from degraded vaccine infection (density)
$\beta_{V}$	Transmission rate of vaccine
$\beta_{W}$	Transmission rate of degraded vaccine
$\gamma_{V}$	Recovery rate of vaccine infection
$\gamma_{W}$	Recovery rate of degraded vaccine infection
b	Birth rate
d	Death rate
σ	Fraction of individuals inoculated with the transmissible vaccine at birth
μ	Mutation rate of the vaccine

**FIGURE 6:**
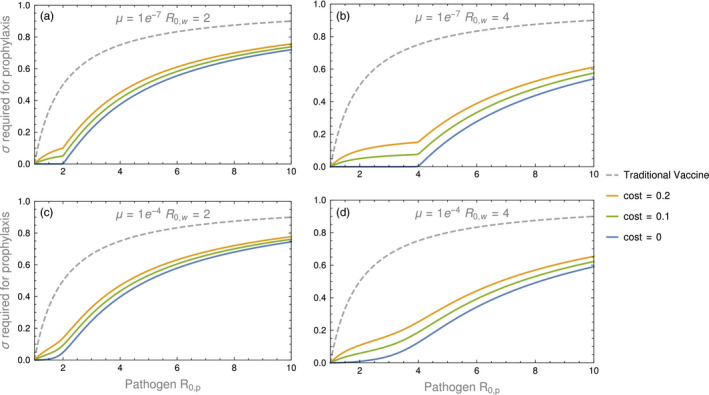
The amount of direct vaccination necessary to protect a population from invasion by a pathogen of different *R*_0_’s (the text indicates how *R*_0_ is calculated from parameters). A mutation‐free vaccine would outgrow the pathogen (and needs no supplementation) up to the point that the pathogen *R*_0_ exceeds the vaccine *R*_0_. Beyond that, increasing levels of supplementation are required to offset higher levels of pathogen *R*_0_. Those effects are purely demographic. The magnitude and effect of vaccine evolution in this system is determined by the mutation rate and selective cost of carrying the transgene. It is seen that vaccine evolution invariably increases the required supplementation, from the effects of both mutation and selection, but the effects can be relatively modest against the background of supplementation required in the absence of evolution. Figure results universally use a birth rate of *b* = 10, a death rate of *d* = 0.01, and a recovery rate of *γ_v_* = 0.1. The cost of carrying the antigen was calculated as 1 − *R*_0_ *_,v_*/*R*_0_ *_,w_* which describes the relative decrease in reproductive number resulting from carrying an antigen. The method of calculating the level of direct vaccination to protect a population against invasion is given in the Appendix 1

Numerical simulation of Equations ((6), (7), (8), (9), (10)) reveals that, relative to a nontransmissible vaccine, the costs of expressing an immunogenic transgene are largely overshadowed by the advantage of even weak vaccine transmission (Figure 6). Furthermore, and unlike in the bioreactor case, the vaccine does not need to infect the entire host population to be effective. Instead, the vaccine only needs to reduce the pool of individuals susceptible to the pathogen below an eradication threshold.

The solid lines in each panel of Figure 6 show the level of supplementation required for prophylaxis (so that the pathogen cannot invade). The blue line (the lowest of the three solid lines) represents the effect of mutation alone, when there is no difference in the *R*_0_ of the vaccine and the vector. For low mutation rates, these blue curves show the intuitive pattern that no (ongoing) supplementation is required so long as the vaccine *R*_0_ exceeds the pathogen *R*_0_—the vaccine merely outgrows the pathogen. Increasing supplementation is required as the vaccine *R*_0_ falls increasingly below the pathogen *R*_0_. This pattern can be inferred from prior work (Basinski et al., 2018) The effect of evolution on supplementation level is seen as the mutation rate increases and as the cost increases from carrying the antigenic insert.

Even if the cost of carrying the antigen is high, the extra amount of direct vaccination needed to achieve pathogen eradication only needs to offset this cost. In other words, if a transmissible vaccine can eliminate the pathogen in the absence of selection and mutation, supplemental direct vaccination only needs to counteract these additional effects to be successful.

## 3 DISCUSSION

Mathematical models developed here investigated repeated introduction (supplementation) as a means of thwarting or mitigating unwanted evolution of genetically engineered systems. Three contexts of genetic engineering were investigated: bioreactors, gene drives, and transmissible vaccines. Supplementation can work to suppress evolution in all systems, but the level required may be impractical in many applications unless selection and mutation are weak.

The bioreactor example is the easiest to illustrate. Two extreme delivery forms of supplementation were considered: continuous supplementation at rate *σ* per generation and entire culture replacement when the transgene frequency fell below a threshold. The required *σ* for maintenance of the transgene at frequency $\hat{p}$ was found to closely approximate $\hat{p}s$ per generation, where *s* is the selective disadvantage of the transgene. For even mild costs of transgene carriage, say 0.03, replacement of 1.5% of the culture volume would be required per generation to maintain the transgene at 50% frequency. This seemingly small level of supplementation could be prohibitive in many settings. Furthermore, the culture used for supplementation may itself be subject to the same evolutionary decay process as is the main vessel. Full culture replacement may have advantages over continual supplementation in some contexts, but the two strategies are not directly comparable: Continual supplementation is calculated as an equilibrium process and thus usually insensitive to the mutation rate, whereas culture replacement is episodic and highly dependent on mutation rate. Indeed, each application we considered is unique; generalities were not evident.

The models described in this study are deterministic. However, there is reason to believe that in some cases, genetic drift may alter our results, even alleviate the problem. Stochastic effects might be a benefit, for example, when bioreactor populations start fixed for the transgene. Unwanted evolution depends on initially rare mutations, which might easily be lost due to the sampling involved in replacing a fraction of the culture each generation. Evolution of resistance to suppression gene drives may also experience stochastic effects due to declining population sizes. Investigation into the ability of supplementation to prevent unwanted evolution in a stochastic framework may therefore represent a promising venue for future studies.

As population genetics processes, our models did not address the nature of beneficial mutations, only their fitness effects; nor did they address the inevitability of many different mutations arising. Transgenic lines can mutate to nonfunctionality through a diverse set of mechanisms, some more beneficial than others. A common type of mutation to fully relieve the burden of carrying the transgene is regulatory: either a deletion of the encoding DNA or a block to its transcription so that no RNA (hence no protein) is produced. Mutations altering the amino acid sequence of a protein might also have some benefit, but they are not as a priori likely to relieve the burden on the cell (unless the transgenic protein is interfering with some host function). A full model of supplementation in our different contexts would consider a spectrum of mutations arising (Böndel et al., 2019; Dittmar, Oakley, Conner, Gould, & Schemske, 2016). Considering the most extreme (most beneficial) mutations merely provides a conservative estimate of the supplementation needed. For example, in the bioreactor model, $\hat{p}s$ is necessarily larger for mutations of greatest benefit. But if evolution had to proceed stepwise through a sequence of individually small mutations, the requisite supplementation might be considerably reduced.

Ultimately, the idea that migration can slow the pace of evolution is not new (Bulmer, 1972; Levene, 1953; Wright, 1940). Despite this, gene flow has only recently been considered as a means to manage the evolution of populations. In particular, a technique known as assisted gene flow uses the directed movement of individuals across a species’ range to hasten local adaptation by introducing favorable traits into populations when and where they might be needed (Aitken & Whitlock, 2013; Kelly & Phillips, 2016; Whiteley, Fitzpatrick, Funk, & Tallmon, 2015). Such an approach can mitigate the effects of maladaptation resulting from rapid environmental change caused by human habitat alteration or climate change.

Less attention has been paid, however, to utilizing gene flow, or supplementation, to slow down or reverse undesirable evolution (Bull & Barrick, 2017). The potential applications are varied, ranging from maintaining the purity of cell lines to preventing the spread of drug and pesticide resistance. This study represents a first step at applying this method to the maintenance of transgenes but, as shown here, arresting evolution using supplementation comes with multifaceted challenges—often requiring a large effort to be successful.

As genetic engineering advances, some of these challenges can be mitigated. By reducing the selective costs imposed by transgenic inserts and increasing their stability, supplementation can be made more feasible. Transgene loss in bioreactors can be slowed by engineering them in linkage to drug‐resistance genes (Rugbjerg, Myling‐Petersen, Porse, Sarup‐Lytzen, & Sommer, 2018). Technologies such as modification gene drives could assist supplementation by biasing segregation in favor of transgenes or by directly modifying the mutation rate (Chavez et al., 2018). In addition to the advantages afforded by these new techniques, the solutions to the problem of unwanted evolution will undoubtedly benefit from the continual re‐introduction of old ideas, offering a concrete foundation with which to ground rapid advances in genetic engineering and biotechnology.

## Supporting information

Data S1

Data S1

Data S1

Data S1

## Data Availability

The data that support the findings of this study are available at the Dryad Digital Repository: https://doi.org/10.5061/dryad.1ns1rn8rp

## APPENDIX 1

The first two models presented are expansions of standard discrete–time population genetics models. An outline of their derivation is presented for both the bioreactor and gene drive models. The vaccine model is an expansion of a previous continuous‐time SIR model, and results were generated using Mathematica 11.3.0.0.

### Maintaining engineered genes in a bioreactor

For the bioreactor model, the life cycle consists of (a) selection, (b) mutation, and (c) supplementation. In the first step, the frequency of the transgene, *p*, is modified by selection to become:

(1) $$\begin{array}{c} p^{\prime }=\frac{W_{A}p}{\bar{W}} \end{array},$$

where $\bar{W}$ is the mean fitness of the population and $W_{A}$ is the fitness of transgene (*A*) bearing individuals. Following selection, mutation degrades some fraction, *μ*, of available transgenes and their frequency becomes:

(2) $$\begin{array}{c} p^{\prime \prime }=p^{\prime }\left(1‐\mu \right) \end{array}.$$

Finally, supplementation replaces a random fraction of the population with individuals drawn from a standardized stock, yielding:

(3) $$\begin{array}{c} p^{\prime \prime \prime }=p^{\prime \prime }\left(1‐\sigma \right)+\sigma \left(1‐\mu \right)=\frac{p\left(1‐s\right)\left(1‐\mu \right)\left(1‐\sigma \right)}{1‐ps} \end{array}.$$

Setting the cumulative change over each generation, $p^{\prime \prime \prime }‐p$, to zero and solving for *σ* yields the amount of supplementation required to maintain steady state (text Equation 2).

### Delaying evolution of resistance to gene drives

The gene drive model utilizes a similar approach with the additional complications of diploidy and biased segregation during meiosis. The life cycle consists of (a) random pairing of gametes to form zygotes, (b) selection, (c) supplementation, (d) reproduction accounting for drive conversion, and (e) mutation. A description of the steps necessary to reproduce Equations (4) and (5) is as follows: First, the union of random gametes is assumed to produce Hardy–Weinberg genotype proportions as shown in the “Frequency” column of Table 3. Following this, the frequencies of each of the six possible genotypes were adjusted based on relative fitness. Next, a portion of the population, *σ*, was replaced with *DW* heterozygotes. Gametes were formed and the frequencies of the driving allele (*D*), the wild‐type allele (*W*), and the resistant allele (*R*) calculated. During this step, a fraction (*c*) of the *W* alleles contributed to the gamete pool by *DW* individuals were converted to *D* by the drive. Finally, mutation converted a portion of *W* to *R*. No simple solutions for equilibria or their stability were found, and analysis proceeded by iterating the resulting recursion equations forward in time.

A similar process with supplementation of the *DD* instead of *DW* genotype produces:

(4) $$\begin{array}{c} w^{\prime }=\frac{w\left(1‐cd‐dhs\left(1‐c\right)\right)\left(1‐\mu \right)\left(1‐\sigma \right)}{1‐ds\left(d+2h\left(1‐d\right)\right)} \end{array},$$

(5) $$\begin{array}{c} r^{\prime }=\frac{\left(r\left(1‐dhs\right)+\mu w\left(1‐cd‐dhs\left(1‐c\right)\right)\right)\left(1‐\sigma \right)}{1‐ds\left(d+2h\left(1‐d\right)\right)} \end{array}.$$

### Supplementation to offset evolution of transmissible vaccines

The level of supplementation with a mutating transmissible vaccine necessary to protect a population against a pathogen can be found by first identifying the equilibrium number of individuals susceptible to the pathogen (*S**) for a given level of supplementation. This was done by solving for the vaccine endemic steady state of text Equations (6)–(10). We assumed that the pathogen was initially absent from the population and capable of infecting any individual not exposed to the vaccine. A pathogen can then only invade if there is a fraction of susceptible individuals greater than 1 divided by the *R*_0_ of the pathogen (*R*_0_ *_,P_*). By setting $\left(\frac{b}{d}\right)\left(\frac{1}{R_{0,P}}\right)=S*$ and solving for sigma, we get the supplementation necessary to just barely keep out a pathogen of a given *R*_0_ ($\sigma_{\text{crit}}$). The attached Mathematica notebook details the steps taken to produce the following:

(6) $$\begin{array}{c} \sigma_{\text{crit}}=\left(1‐\frac{1}{R_{0,P}}\right)\left(1‐\frac{KR_{0,V}}{2R_{0,P}}\right)\left(\frac{d+\gamma_{V}+\mu }{d+\gamma_{V}}\right), \end{array}$$

with *K* defined as

(7) $$\begin{array}{c} K=1+\frac{R_{0,P}}{R_{0,W}}‐\sqrt{{\left(\frac{R_{0,P}‐R_{0,W}}{R_{0,W}}\right)}^{2}+\frac{4\mu R_{0,P}\left(R_{0,P}‐1\right)}{R_{0,W}\left(d+\gamma_{V}\right)}} \end{array}.$$
