# Supplementary material for: Suppressing evolution in genetically engineered systems through repeated supplementation
Source: Evol Appl. 2020 Nov 6;14(2):348–59. doi: 10.1111/eva.13119 (PMC7896713; doi:10.1111/eva.13119)
Supplement: Supplementary file 3 — Data S1 [file EVA-14-348-s003.docx]

Suppressing evolution in genetically engineered systems through repeated supplementation.

Layman, N. C.^1,*^, Tuschhoff, B. M.^2^, Basinski, A. J.^3^, Remien, C. H.^4^, Bull, J. J.^5^, and S. L. Nuismer^6^.

**Author Contact Info:**

^1,*^[nlayman@uidaho.edu](mailto:nlayman@uidaho.edu), ^2^[tusc3130@vandals.uidaho.edu](mailto:tusc3130@vandals.uidaho.edu), ^3^[abasinski@uidaho.edu](mailto:abasinski@uidaho.edu),

^4^[cremien@uidaho.edu](mailto:cremien@uidaho.edu), ^5^[jbull@uidaho.edu](mailto:jbull@uidaho.edu), ^6^[snuismer@uidaho.edu](mailto:snuismer@uidaho.edu)

**Running Title:** Swamping and the genetic stability of genetically modified organisms.

**Keywords:** Swamping, gene flow, genetic engineering, gene drive, bioreactor, transmissible vaccine

**Acknowledgments:** The authors would like to acknowledge Tanner Varrelman, Mark Smithson and Enrique J. Schwarzkopf for their valuable feedback. This work was funded by NIH grant R01GM122079 (S.L.N.)

^*^Corresponding author

^1,5,6^Department of Biological Sciences, University of Idaho, Moscow ID, 83844

^2,3,4^Department of Mathematics, University of Idaho, Moscow ID, 83844
